# Optimal timing of endoscopic retrograde cholangiopancreatography for acute cholangitis associated with distal malignant biliary obstruction

**DOI:** 10.1186/s12876-021-01755-z

**Published:** 2021-04-17

**Authors:** Namyoung Park, Sang Hyub Lee, Min Su You, Joo Seong Kim, Gunn Huh, Jung Won Chun, In Rae Cho, Woo Hyun Paik, Ji Kon Ryu, Yong-Tae Kim

**Affiliations:** Division of Gastroenterology, Department of Internal Medicine, Liver Research Institute, College of Medicine, Seoul National University Hospital, Seoul National University, 101, Daehak-ro, Jongno-gu, Seoul, 03080 Republic of Korea

**Keywords:** Cholangitis, Endoscopic retrograde cholangiopancreatography, Neoplasms, Early intervention, Treatment outcomes

## Abstract

**Background:**

There is a lack of studies regarding the optimal timing for endoscopic retrograde cholangiopancreatography (ERCP) in patients with cholangitis caused by distal malignant biliary obstruction (MBO). This study aims to investigate the optimal timing of ERCP in patients with acute cholangitis associated with distal MBO with a naïve papilla.

**Methods:**

A total of 421 patients with acute cholangitis, associated with distal MBO, were enrolled for this study. An urgent ERCP was defined as being an ERCP performed within 24 h following emergency room (ER) arrival, and early ERCP was defined as an ERCP performed between 24 and 48 h following ER arrival. We evaluated both 30-day and 180-day mortality as primary outcomes, according to the timing of the ERCP.

**Results:**

The urgent ERCP group showed the lowest 30-day mortality rate (2.2%), as compared to the early and delayed ERCP groups (4.3% and 13.5%) (*P* < 0.001). The 180-day mortality rate was lowest in the urgent ERCP group, followed by early ERCP and delayed ERCP groups (39.4%, 44.8%, 60.8%; *P* = 0.006). A subgroup analysis showed that in both the primary distal MBO group, as well as in the moderate-to-severe cholangitis group, the urgent ERCP had significantly improved in both 30-day and 180-day mortality rates. However, in the secondary MBO and mild cholangitis groups, the difference in mortality rate between urgent, early, and delayed ERCP groups was not significant.

**Conclusions:**

In patients with acute cholangitis associated with distal MBO, urgent ERCP might be helpful in improving the prognosis, especially in patients with primary distal MBO or moderate-to-severe cholangitis.

**Supplementary Information:**

The online version contains supplementary material available at 10.1186/s12876-021-01755-z.

## Background

Acute cholangitis is an infection of biliary system as a result of biliary stasis [1]. This can be life-threatening without timely intervention, such as biliary drainage and adequate antibiotics [1–5]. Early endoscopic retrograde cholangiopancreatography (ERCP) done within 48 h in patients with moderate-to-severe cholangitis is known to reduce the duration of hospitalization, mortality rates, and adverse events, such as multiple organ failure [3–7]. The most common cause of obstruction is choledocholithiasis, which accounts for about half of the cases [7–9]. Malignant biliary obstruction (MBO), such as pancreatic cancer, cholangiocarcinoma, or metastatic cancer, constitutes 10–30% of cholangitis cases [3, 5, 7–9].

Patients with biliary stones and those with MBO show different clinical courses and prognoses. Cholangitis caused by biliary stones can be definitely treated by biliary drainage and removing the stones [10]. On the other hand, patients with malignant biliary stricture require additional treatment for the underlying disease following adequate biliary decompression. Because patients with MBO are usually treated with chemotherapy for the underlying disease, most patients have poor oral intake, poor performance status, and are susceptible to infection [11–13]. According to a recent study of patients with acute cholangitis by Parikh et al. [14] MBO is associated with a higher risk of readmission within 30 days. MBO is often combined with anatomical alteration in the gastrointestinal tract, which can make insertion and cannulation difficult [15, 16]. The stage of cancer also affects the prognosis. Unlike patients with early-stage cancer, which can be treated curatively, the prognosis for patients with MBO caused by metastatic cancer is worse, regardless of adequate biliary drainage [17].

Previous studies emphasizing the role of early ERCP have been based on populations with heterogeneous etiologies and mostly included patients with biliary stones [8, 18, 19]. In a study by Tan et al. [19] 45% of all cholangitis was caused by common bile duct stones, and 43% by MBO. Another study by Kiriyama et al. [8] is based on patients with various etiologies. The current Tokyo Guidelines recommend early or urgent ERCP, depending on the severity, but they do not mention the etiology [20].

To date, there is a lack of studies regarding the optimal timing for ERCP in patients with cholangitis caused by distal MBO. The purpose of this study is to evaluate the outcomes according to the timing of ERCP in patients with acute cholangitis due to distal MBO.

## Methods

### Patients and enrollment criteria

**Fig. 1:**
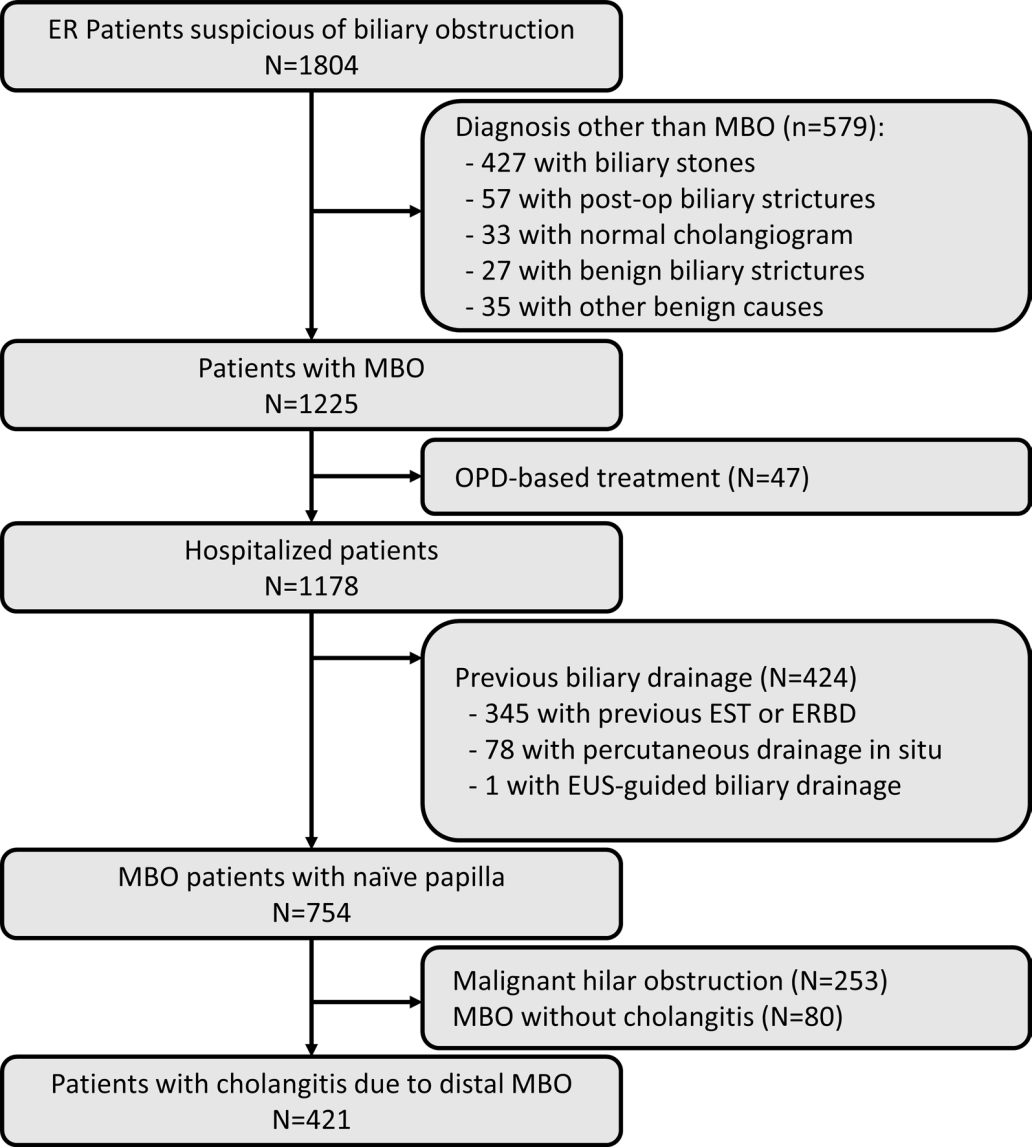
Flowchart of patient enrollment

This is a retrospective study at Seoul National University Hospital. From January 2005 to June 2018, we analyzed 1,804 patients who had visited the emergency room (ER) and had undergone ERCP for suspected biliary obstruction. The 10th edition of the International Classification of Diseases (ICD-10) codes and pathologic reports were reviewed to identify patients with MBO. Patients with biliary stones or other benign etiologies were excluded. Patients who had been discharged from the ER right after ERCP and had received outpatient department-based treatment were also excluded. We identified 754 patients as MBO patients with a naïve papilla after we had excluded those who had previously undergone endoscopic sphincterotomy, percutaneous transhepatic biliary drainage, or endoscopic ultrasound-guided biliary drainage. After excluding patients with hilar obstruction and those without cholangitis, a total of 421 patients were included (Fig. 1). This study was approved by the Institutional Review Board of Seoul National University Hospital, Seoul, Korea (1802-123-924).

### Data collection and definitions

Demographic data, including age, sex, and comorbidities was collected. Patient complaints were investigated at the time of ER arrival, and vital signs, including blood pressure, body temperature, respiratory rate, heart rate, and oxygen saturation were measured. Laboratory findings, including white blood cell count, platelet count, C-reactive protein, total bilirubin, albumin, aspartate transaminase, alanine transaminase, alkaline phosphatase, gamma glutamyl transpeptidase, creatinine, and prothrombin time were obtained. If, based on the patient’s symptoms and laboratory findings, the biliary tract obstruction was suspicious, computed tomography scans covering the biliary tree were performed. The diagnosis of MBO was based on the obstructive patterns of the liver function test, concurrent with bile duct strictures on the imaging findings. Cholangitis was defined and graded according to the 2018 Tokyo Guidelines [20]. Previous anticancer treatments, such as curative surgery, chemotherapy, or radiation therapy were investigated. Following ERCP, the cancer status was assessed, and treatment types, including surgery and palliative chemotherapy, were investigated. Death data was collected from the Korean Ministry of the Interior and Safety database.

Urgent ERCP was defined as ERCP performed within 24 h, and early ERCP was defined as ERCP performed between 24 to 48 h following ER arrival [20]. ERCP performed after 48 h was defined as delayed ERCP [21]. The physicians decided when to perform ERCP based on the condition of patients including the moderate to severe cholangitis. MBO was classified as primary and secondary distal MBO, according to the origin of biliary obstructive lesion. Primary distal MBO was defined as a cancer originally located in the pancreatic head, bile duct, ampulla of Vater, periampullary duodenum, or the gallbladder directly invading the mid-to-distal CBD; whereas secondary distal MBO was defined as any other cancer with metastasis to the peribiliary lymph nodes and soft tissues.

### Procedures

All ERCPs were performed under moderate sedation using midazolam and meperidine. Side-viewing duodenoscopes were used (TJF-260, JF-260, TJF-240, JF-240, TJF-200, and JF-200; Olympus Optical, Tokyo, Japan). After cannulation of the ampulla of Vater, sphincterotomy was performed at the discretion of the performing clinicians. Contrast was injected after the cannulation, and fluoroscopic findings were obtained to evaluate the biliary tree. Once the obstructive level had been identified, the clinician passed guidewire to the upstream part of the bile duct obstruction, and inserted a plastic stent and/or self-expandable metal stent. If a tight biliary stricture was expected, the stent was inserted following balloon dilatation at the stricture site.

### Study outcome measures

The primary study outcomes were the 30-day mortality rate and the 180-day mortality rate between those patients who had undergone urgent, early or delayed ERCP. Secondary outcomes were the technical success rate, the clinical success rate, differences in hospital stay, and postprocedural adverse events, such as pancreatitis, bleeding, and perforation. Technical success was defined as a successful deployment of biliary stents at the stricture site on the first ERCP. Clinical success was defined as ≥ 30% decrease in total bilirubin over a four-week period [22]. Post-ERCP adverse events were based on the lexicon guidelines of the American Society for Gastrointestinal Endoscopy [23].

### Statistical analysis

Continuous variables were expressed as a mean (± standard deviation) or a median (with interquartile range), and categorical variables as numbers and percentages. Student’s t test, pairwise Wilcoxon rank sum test, or Kruskal–Wallis rank sum test was used for comparison of continuous variables between the groups. Pearson’s chi-square test or Fisher’s exact test was used for comparison of categorical value. Post hoc analysis was performed using the Bonferroni method. In addition, a multivariable analysis was conducted to assess the possible risk factors for primary outcomes with *p*-values ≤ 0.1 in the univariable analysis and to adjust for age, sex and ER visit during holidays. Akaike Information Criterion-based backward selection was used in the multivariable logistic regression analysis [24]. All statistical analyses were conducted using the R s/w environment (version 3.6.3; The R Foundation for Statistical Computing, Vienna, Austria), and a *P*-value < 0.05 was considered to be statistically significant.

## Results

### Baseline characteristics

Table 1 summarizes the baseline characteristics. Urgent, early, and delayed ERCPs were performed in 231 (54.9%), 116 (27.6%) and 74 (17.6%) patients, respectively. The median time from ER arrival to ERCP was 24.0 (13.2–44.7) hours for mild (*n* = 86), 22.2 (7.2–30.6) hours for moderate (*n* = 289), and 25.9 (18.4–43.4) hours for severe (*n* = 46) grade of cholangitis, and there was no significant difference between them (*P* = 0.087). Prior to the initial ERCP, 135 (32.1%) and 52 (12.4%) patients had received either palliative systemic chemotherapy or curative resection for underlying malignancy, respectively. Among 421 patients, technical success was achieved in 348 patients. Plastic stents were used for 173 patients and metal stents were used for 175 patients. Twelve patients required balloon dilatation for stent insertion due to tight biliary stricture, while no patient received bougination for their biliary stricture.

**Table 1:** Baseline characteristics

Variables	(N = 421)
Age	67.0 (59.0–73.0)
Sex	
Male	251 (59.6%)
Female	170 (40.4%)
Etiology of MBO	
Biliary tract	184 (43.7%)
Pancreas	150 (35.6%)
Upper GI tract	35 (8.3%)
Lower GI tract	21 (5.0%)
Lung	10 (2.4%)
Genitourinary tract	8 (1.9%)
Breast	6 (1.4%)
Liver	5 (1.2%)
Others	2 (0.5%)
Underlying disease	
Hypertension	192 (45.6%)
Diabetes mellitus	99 (23.5%)
Cardiovascular disease	32 (7.6%)
Chronic liver disease	26 (6.2%)
Chronic kidney disease	5 (1.2%)
Chronic obstructive pulmonary disease	10 (2.4%)
Primary MBO	341 (81.0%)
Cholangitis severity^a^	
Mild	86 (20.4%)
Moderate	289 (68.6%)
Severe	46 (10.9%)
Previous upper GI surgery	15 (3.6%)
Initial laboratory findings	
White blood cell, 10^3^/µL	8.0 ± 3.8
Platelet, 10^3^/μL	245.2 ± 103.1
CRP, mg/dL	5.8 ± 5.7
Creatinine, mg/dL	0.8 ± 0.6
Albumin, g/dL	3.2 ± 0.5
Total bilirubin, mg/dL	7.9 ± 6.4
AST, IU/L	122.1 ± 138.9
ALT, IU/L	146.8 ± 134.5
ALP, IU/L	461.4 ± 306.0
GGT, IU/L	674.7 ± 518.8
PT (INR)	1.1 ± 0.2

*MBO* malignant biliary obstruction, *GI* gastrointestinal, *CRP* C-reactive protein, *AST* aspartate transaminase, *ALT* alanine transaminase, *ALP* alkaline phosphatase, *GGT* gamma glutamyl transpeptidase, *PT* prothrombin time, *INR* international normalized ratio

^a^Was defined according to the Tokyo Guidelines of 2018

The median time before the procedure was 23.1 (8.4–33.0) hours, 11.8 (5.5–20.9) hours in urgent group, 28.6 (25.9–36.4) hours in early group, and 70.9 (53.5–98.8) hours in the delayed ERCP group (*P* < 0.001). There were no significant differences in age, sex, primary cancer location, Charlson’s Comorbidity Index (CCI), the proportion of primary MBO, cholangitis severity, the type of biliary stent, and the need for balloon dilatation between the three groups. Table 2 summarizes the differences in baseline characteristics between the patients in the urgent, early, and the delayed ERCP groups.

**Table 2:** Differences in baseline characteristics between the urgent, early, and elective ERCP groups

Variables	Urgent ERCP (N = 231)	Early ERCP (N = 116)	Delayed ERCP (N = 74)	*P* value
Age	67.0 (59.0–73.0)	67.0 (60.0–73.0)	66.0 (59.0–72.0)	0.967
Sex				0.858
Male	139 (60.2%)	70 (60.3%)	42 (56.8%)	
Female	92 (39.8%)	46 (39.7%)	32 (43.2%)	
CCI	5.5 ± 1.7	5.5 ± 1.8	5.9 ± 1.9	0.223
Primary MBO	189 (81.8%)	96 (82.8%)	56 (75.7%)	0.428
Cholangitis severity^a^				0.536
Mild	43 (18.6%)	25 (21.6%)	18 (24.3%)	
Moderate to severe	188 (81.4%)	91 (78.4%)	56 (75.7%)	
Previous upper GI surgery	10 (4.3%)	4 (3.4%)	1 (1.4%)	0.484
ER arrival at holidays^b^	45 (19.5%)	37 (31.9%)	48 (64.9%)	< 0.001

*ERCP* endoscopic retrograde cholangiopancreatography, *MBO* malignant biliary obstruction, *GI* gastrointestinal, *CCI* Charlson’s comorbidity index, *ER* emergency room

^a^Was defined according to the 2018 Tokyo Guidelines

^b^Also included the day before holidays

### The differences in 30-day and 180-day mortality rates between each study group

**Fig. 2:**
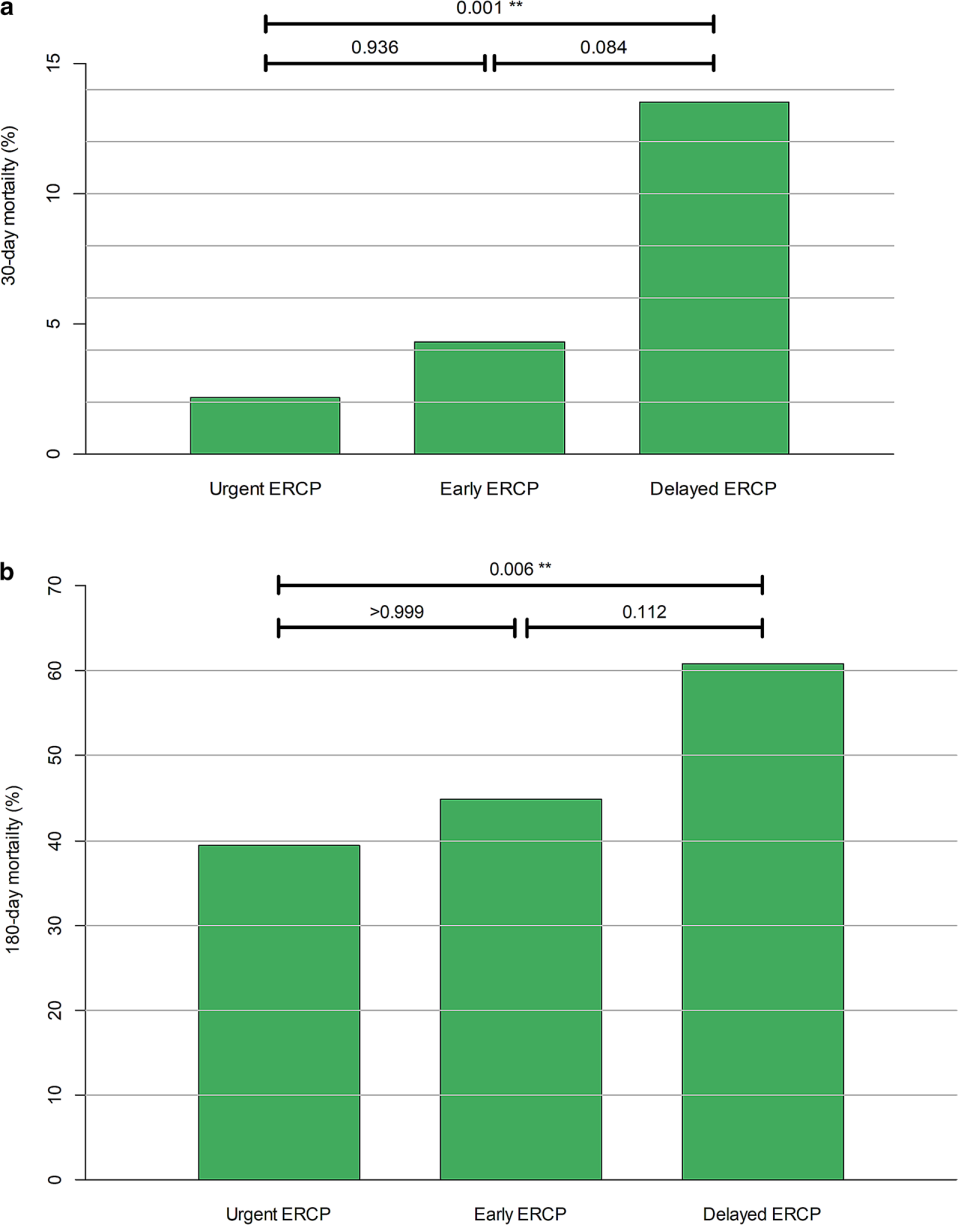
The difference in 30-day mortality rate (**a**) and 180-day mortality rate (**b**) between urgent, early and delayed ERCP groups

The 30-day mortality rate was lowest in the urgent ERCP group, followed by the early and delayed ERCP groups (2.2%, 4.3%, 13.5%; *P* < 0.001). In the post hoc analysis, the 30-day mortality in the urgent ERCP group was significantly lower than in the delayed ERCP group (*P* = 0.001), whereas the difference between early and delayed ERCP groups was not significant (*P* = 0.084). The differences in the 180-day mortality rate between the urgent, early, and delayed ERCP groups were also significant (39.4%, 44.8%, 60.8%; *P* = 0.006). In the post hoc analysis, the urgent ERCP group showed a significantly lower mortality rate as compared to the delayed ERCP group (*P* = 0.006), but the early ERCP group did not (*P* = 0.112). Figure 2 demonstrates the differences in both the 30-day and 180-day mortality rates in each study group.

Table 3 shows the result of a multivariable analysis, which revealed that urgent ERCP (OR 0.11 [0.03–0.34]; *P* < 0.001), early ERCP (OR 0.23 [0.07–0.75]; *P* = 0.015) and secondary MBO (OR 3.33 [1.22–9.06]; *P* = 0.019) are associated with a 30-day mortality rate. Table 4 demonstrates that a 180-day mortality rate is significantly associated with urgent ERCP (OR 0.41 [0.23–0.72]; *P* = 0.002), early ERCP (OR 0.54 [0.29–1.00]; *P* = 0.049), and secondary MBO (OR 4.39 [2.52–7.63]; *P* < 0.001) in the multivariable analysis.

**Table 3:** Univariable and multivariable analysis identifying factors related to 30-day mortality

	Univariable		Multivariable	
	Odds ratio (95% CI)	*P* value	Odds ratio (95% CI)	*P* value
Age ≥ 75	0.40 (0.04–1.70)	0.270		
Male sex	2.83 (0.93–8.60)	0.095	2.88 (0.90–9.20)	0.074
Timing of ERCP				
> 48 h			1 (Reference)	
24–48 h		0.084	0.23 (0.07–0.75)	0.015
< 24 h		0.001	0.11 (0.03–0.34)	< 0.001
Secondary MBO	3.04 (1.04–8.42)	0.035	3.33 (1.22–9.06)	0.019
Mild cholangitis	0.00 (0.00–0.76)	0.019	0.00 (0.00-infinity)	0.987
ER arrival at holidays	1.52 (0.61–3.82)	0.511		

ERCP, endoscopic retrograde cholangiopancreatography; MBO, malignant biliary obstruction, ER: emergency room

**Table 4:** Univariable and multivariable analysis identifying factors related to 180-day mortality

	Univariable		Multivariable	
	Odds ratio (95% CI)	*P* value	Odds ratio (95% CI)	*P* value
Age ≥ 75	0.62 (0.38–1.00)	0.066	0.61 (0.36–1.02)	0.060
Male sex	0.96 (0.65–1.42)	0.907		
Timing of ERCP				
> 48 h			1 [Reference]	
24–48 h		0.112	0.54 (0.29–1.00)	0.049
< 24 h		0.006	0.41 (0.23–0.72)	0.002
Secondary MBO	4.62 (2.68–7.95)	< 0.001	4.39 (2.52–7.63)	< 0.001
Mild cholangitis	0.72 (0.45–1.17)	0.233	0.59 (0.35–1.01)	0.056
ER arrival at holidays	0.91 (0.60–1.38)	0.742		

*ERCP* endoscopic retrograde cholangiopancreatography, *MBO* malignant biliary obstruction, *ER* emergency room

Neither the type of biliary stent (*P* = 0.145) nor the need for biliary dilation (*P* = 0.560) were significantly associated with the 30-day mortality rate. However, the use of metal stent for biliary drainage was associated with the increased 180-day mortality rate (OR 9.99 [6.04–16.52]; *P* < 0.001), while the need for balloon dilatation was not significantly associated with a 180-day mortality rate (OR 1.76 [0.55–5.65]; *P* = 0.340).

Among 20 patients who died within 30 days after ERCP, there were 12 patients whose cause of death could be identified. The most common cause of death was disease progression (8 patients, 66.7%), followed by infection other than cholangitis (3 patients, 25%), and uncontrolled benign gastric ulcer bleeding (1 patient, 8.3%). The cause of 180-day mortality, however, was hardly identifiable. In most of patients who died within 180 days after ERCP, we were not able to determine the cause of death (137 patients, 72.9%).

### Subgroup analysis based on the etiology and severity of cholangitis

In the subgroup analysis, the difference in the 30-day mortality rates between the urgent, early, and delayed ERCP groups was significant in the primary MBO group (1.6%, 3.1%, 10.7%; *P* = 0.007, Additional file 1) and in the moderate-to-severe cholangitis group (2.7%, 5.5%, 17.9%; *P* < 0.001). The 180-day mortality rates also showed significant differences between the urgent, early, and delayed ERCP groups in the primary MBO patients (31.7%, 40.6%, 53.6%; *P* = 0.010, Additional file 2) and the moderate-to-severe cholangitis patients (39.9%, 49.5%, 62.5%; *P* = 0.009). In the post hoc analysis, the difference between the urgent and delayed ERCP groups was significant in all subgroups. Early ERCP, however, did not show any significant differences from delayed ERCP in all subgroups. Patients with secondary MBO or mild cholangitis did not show significant differences in the 30-day (secondary MBO: 4.8%, 10.0% 22.2%; *P* = 0.094, mild cholangitis: 0.0%, 0.0%, 0.0%, *P* > 0.999) and the 180-day mortality rates within all study groups (secondary MBO: 73.8%, 65.0% 83.3%; *P* = 0.468, mild cholangitis: 37.2%, 28.0%, 55.6%; *P* = 0.182).

### Secondary outcomes according to the timing of ERCP

Table 5 summarizes the differences in secondary outcomes among the three study groups classified by ERCP timing. The overall technical success rate was 82.7%, and the differences between each ERCP group were not significant (*P* = 0.120). The clinical success rate in the overall cohort was 75.1%, and the urgent ERCP group showed the highest clinical success rate, followed by the early and delayed ERCP groups (80.1%, 75.0% and 59.5%; *P* = 0.002). Adverse events related to the procedure were observed in 31 patients (7.4%); pancreatitis in 22 (5.2%), bleeding in 8 (1.9%), and perforation in 1 (0.2%) patient. There were no significant differences in procedure-related adverse events between any of the study groups. Overall, the mean hospital stay was 12.1 ± 19.2 days, and there were no significant differences in any of the ERCP groups (*P* = 0.337).

**Table 5:** Secondary outcomes between three groups classified by duration to ERCP

	Urgent ERCP (N = 231)	Early ERCP (N = 116)	Delayed ERCP (N = 74)	*P* value
Success rate				
Technical	198 (85.7%)	94 (81.0%)	56 (75.7%)	0.120
Clinical	185 (80.1%)	87 (75.0%)	44 (59.5%)	0.002
Post-ERCP adverse events				
Pancreatitis	13 (5.6%)	5 (4.3%)	4 (5.4%)	0.871
Bleeding	5 (2.2%)	3 (2.6%)	0 (0.0%)	0.404
Perforation	1 (0.4%)	0 (0.0%)	0 (0.0%)	0.662
Hospital stay	11.1 ± 22.1	12.1 ± 12.7	14.9 ± 18.1	0.337

*ERCP* endoscopic retrograde cholangiopancreatography

## Discussion

There is limited data evaluating the optimal timing of ERCP in patients with cholangitis caused by distal MBO. In this study, we analyzed the outcomes of urgent or early ERCP in acute cholangitis due to distal MBO. This study found that urgent ERCP clearly improved the 30-day mortality rate and the 180-day mortality rate, especially in patients with primary MBO and moderate-to-severe cholangitis.

Even without considering the etiology, the optimal timing of ERCP in patients with acute cholangitis remains controversial. In a recent meta-analysis, in-hospital mortality was reduced when ERCP had been performed within 24 h as compared to after 24 h, within 48 h as compared to after 48 h, within 72 h as compared to after 72 h [25]. Another recent meta-analysis also demonstrated the beneficial effect of urgent or early ERCP on 30-day mortality [4]. These studies, however, were not able to separate the survival benefits to patients who had received urgent ERCP and early ERCP, because the mortality data for patients who had undergone ERCP between 24 and 48 h was reported inconsistently. We divided this study group into three independent groups according to the timing of the ERCP, and we selected the 30-day and 180-day mortality rates as the primary outcomes, taking into account the subsequent treatments such as chemotherapy and curative surgery. The urgent ERCP group showed a significant improvement in both the 30-day mortality rate and 180-day mortality rate over the delayed ERCP group. Meanwhile, the differences in the 30-day and 180-day mortality rates between early and delayed ERCP groups were not statistically significant. This may be because of the nature of early ERCP but may also be because of an insufficient sample size.

In the Tokyo Guidelines, urgent or early ERCP is recommended for patients with moderate-to-severe cholangitis, and a large-scale observational study showed that urgent and early ERCP improved 30-day mortality in patients with moderate cholangitis [8]. In the case of mild cholangitis, however, biliary drainage should be considered when the initial supportive treatment shows insufficient response [20, 26]. Our study found that the urgent ERCP improved the 30-day mortality rate, as well as the 180-day mortality rate, in moderate-to-severe cholangitis due to distal MBO. This implies that urgent ERCP can increase short-term and medium-term survival rates in the moderate-to-severe cholangitis group by minimizing disruption of subsequent treatment, including curative surgery and palliative chemotherapy for underlying malignant disease. Meanwhile, this survival benefit was limited in patients with mild cholangitis.

In this study, urgent ERCP improved the 30-day and 180-day mortality rates in the primary distal MBO group, whereas the differences of 30-day and 180-day mortality rates were not significant in the secondary distal MBO group. This indicates that urgent ERCP in the primary distal MBO group can improve both the short-term and medium-term outcomes by enabling a timely implementation of subsequent anticancer treatment, such as curative surgery and chemotherapy. In the secondary distal MBO group, however, these anticancer treatment attempts were difficult, even though biliary drainage was successful.

Because of anatomical alteration associated with tumor invasion or previous surgery, endoscopic access to the bile duct can be difficult in patients with distal MBO [15, 16, 27]. In a study of patients with distal MBO due to pancreatic head cancer, adequate drainage was successful in 75% of the cases in the first ERCP trial [28]. Although some randomized controlled trials reported a technical success rate of 90% or higher, few or no patients with a history of previous upper gastrointestinal surgery were included, and no information on previous ERCP history was available [27, 29]. In the present study, the technical success rate was not inferior to previous studies, considering that some of the included patients had anatomical alterations due to previous surgery, and all patients with a previous ERCP history were excluded.

In this study, patients who visited ER on holidays tended to receive ERCP later, because the timing of ERCP was determined by each physician. In univariable and multivariable analysis, however, there were no significant differences in 30-day and 180-day mortality rates between those who visited on weekdays and weekends. This indicates that patients who needed the urgent biliary drainage were treated appropriately by the doctor’s decision, regardless of the day of the hospital visit.

The doctors in this study preferred plastic stents when there was a possibility of curative treatment, and used metal stents when palliative chemotherapy or supportive care was expected. This tendency may affect the better 180-day mortality rate in plastic stent group.

There are several limitations in this study. First, this retrospective, single-center study may have inherent selection bias. Second, about half of the patients (54.9%) underwent urgent ERCP following ER arrival, resulting in a smaller sample size of early and delayed ERCP groups. Caution needs to be applied when interpreting the results of subgroup analysis. Third, since more than half of the patients had not been diagnosed with cancer before ER arrival, comprehensive investigation of the Eastern Cooperative Oncology Group performance status could not be made at the time of ERCP. Instead, this study evaluated the CCI, and provided information on the underlying disease status. Fourth, a variety of carcinomas were included in each group. Staging systems, treatment protocols, and prognoses are largely different for each cancer. This heterogeneity may serve as a confounding factor in outcomes analysis. Fifth, some clinical information, such as the cause of death, could not be analyzed because this was not accessible in the government data. Nevertheless, this study has the strength that it demonstrates therapeutic outcomes of urgent ERCP for acute cholangitis in patients with only distal MBO.

## Conclusions

In conclusion, urgent ERCP might be performed actively in acute cholangitis caused by distal MBO considering the severity and etiology of the MBO. Well-designed larger prospective randomized controlled trials are needed to evaluate the optimal timing of ERCP in patients with acute cholangitis due to distal MBO.

## Supplementary Information

**Additional file 1.** The difference of 30-day mortality rate between urgent, early, and delayed ERCP groups in each subgroup.

**Additional file 2.** The difference of 180-day mortality rate between urgent, early, and delayed ERCP groups in each subgroup.

## Data Availability

The datasets used and/or analyzed during the current study are available from the corresponding author upon reasonable request.

## Abbreviations

ERCP: Endoscopic retrograde cholangiopancreatography
MBO: Malignant biliary obstruction
ER: Emergency room
ICD-10: 10th edition of the International Classification of Diseases
CCI: Charlson’s Comorbidity Index
