# Supplementary figures and images for: Optimal timing of endoscopic retrograde cholangiopancreatography for acute cholangitis associated with distal malignant biliary obstruction
Source: BMC Gastroenterol. 2021 Apr 17;21:175. doi: 10.1186/s12876-021-01755-z (PMC8052855; doi:10.1186/s12876-021-01755-z)

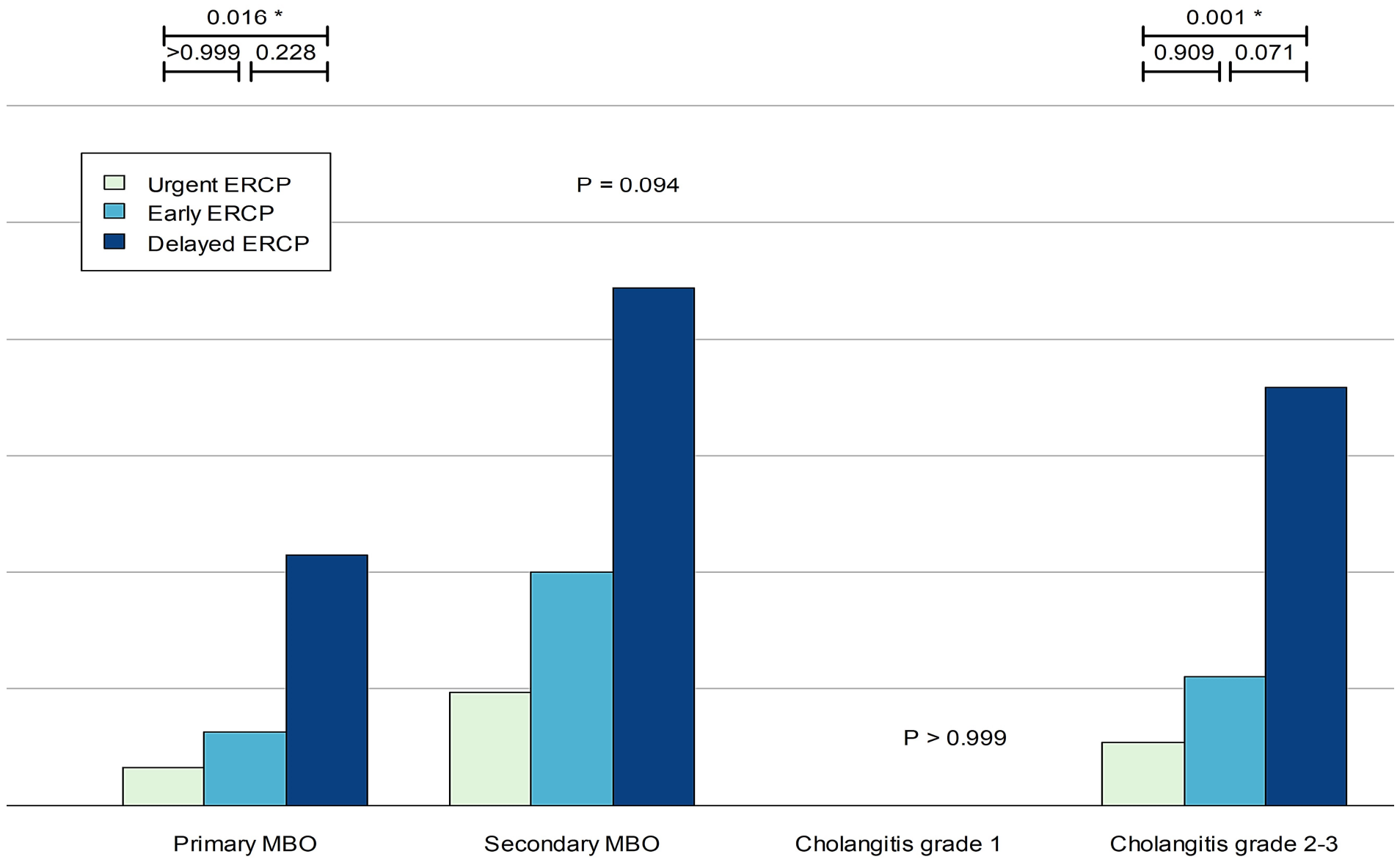

Supplement: Supplementary file 1 — Additional file 1. The difference of 30-day mortality rate between urgent, early, and delayed ERCP groups in each subgroup. [file 12876_2021_1755_MOESM1_ESM.pdf]

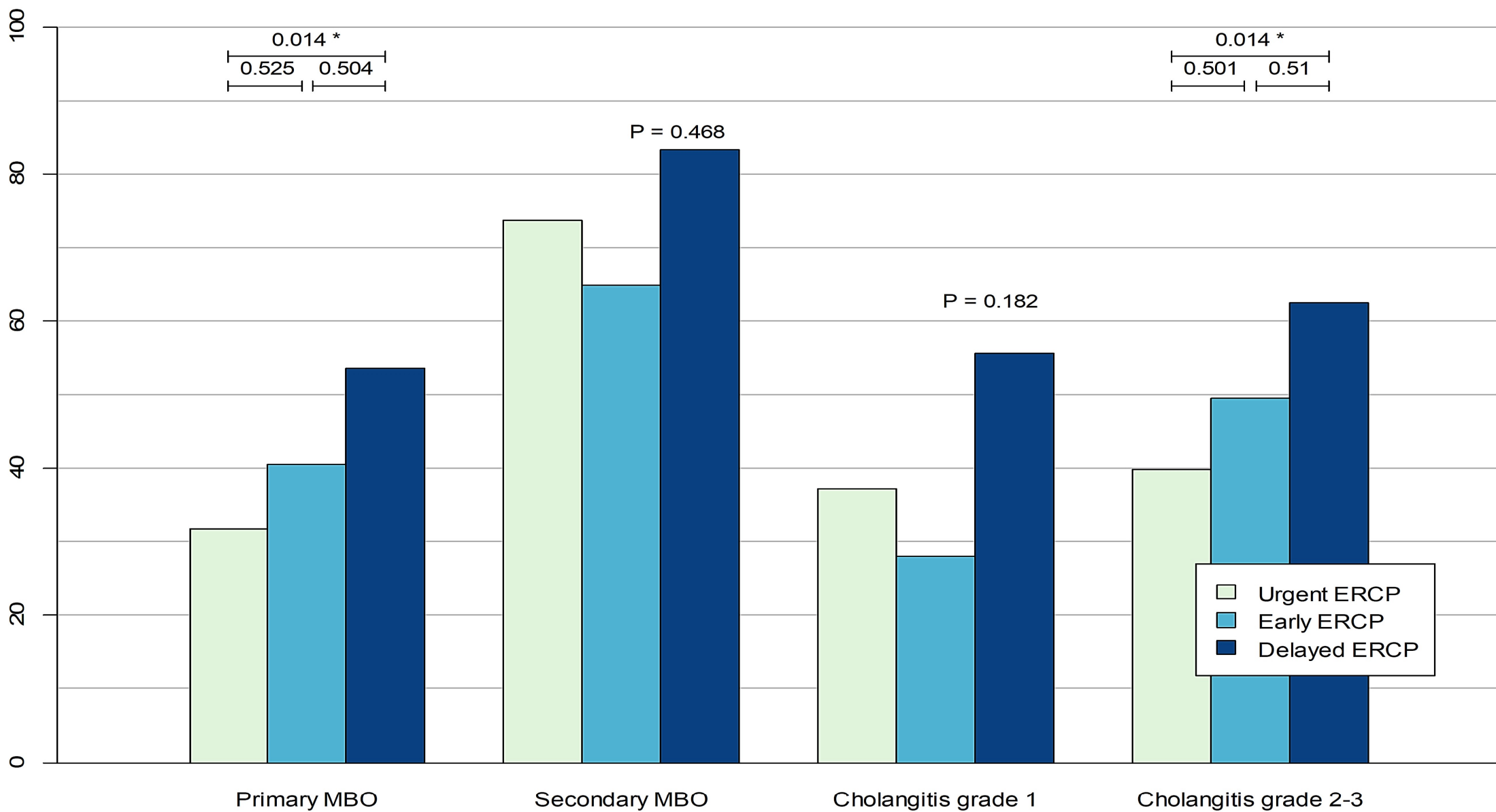

Supplement: Supplementary file 2 — Additional file 2. The difference of 180-day mortality rate between urgent, early, and delayed ERCP groups in each subgroup. [file 12876_2021_1755_MOESM2_ESM.pdf]
